# Isolation and identification of a chicken-derived *Salmonella* phage VB-SalS-XND03, Its biological characteristics and application effect against *Salmonella* infection in chicks

**DOI:** 10.1016/j.psj.2026.107489

**Published:** 2026-07-25

**Authors:** Qirui Zang, Mingshuai Chen, Yingchao Li, Chenyang Shi, Yaolong Song, Ying Huang, Xueqin Lan, Wanjing Jin, Yixin Bai, Gulifeire Yilizhati, Yishan Sun, Shuhui Yang, Yan Wang, Yaqi Huang, Yuan Tian, Wenxing Jiang, Xiaofeng Zheng, Mengfei Zhang, Jinxin Xie, Panpan Tong

**Affiliations:** a College of Veterinary Medicine, Xinjiang Agricultural University, Urumqi, 830052, China; b Xinjiang Key Laboratory of New Drug Research and Development for Herbivores, Urumqi, 830052, China

**Keywords:** *Salmonella*, Bacteriophage, Lytic phage, Biological characteristics, Chick

## Abstract

**Introduction:**

Salmonellosis, a zoonotic disease caused by *Salmonella* spp., poses a major threat to poultry production and public health. The increasing prevalence of antimicrobial resistance has reduced the effectiveness of conventional antibiotics, highlighting the need for alternative strategies such as phage therapy.

**Methods:**

A lytic phage, VB-SalS-XND03, was isolated using multidrug-resistant *S. Enteritidis* J3 as the host strain. Its morphology, host range (43 strains), biological characteristics, and whole-genome sequence were characterized. To mimic the natural fecal-oral transmission route, prophylactic and therapeutic efficacy was evaluated in a healthy chick oral gavage model (n = 8 per group for survival analysis; n = 20 per group for mechanistic studies) using survival analysis, quantitative polymerase chain reaction (qPCR) for inflammatory cytokines and tight junction proteins, histopathology, 16S rRNA sequencing (n = 4/group), transcriptomics (n = 3/group), and metabolomics (n = 3/group). An intraperitoneal challenge model was also included as supplementary validation.

**Results:**

Transmission electron microscopy showed that VB-SalS-XND03 possesses an icosahedral morphology and belongs to the class *Caudoviricetes*, genus *Tequintavirus*. Host range analysis demonstrated serotype-preferential lytic activity, lysing 10 of 43 *Salmonella* strains (23%), predominantly *Salmonella Enteritidis*. The phage exhibited robust environmental stability (pH 4–12, temperature 4–60 °C) and a rapid lytic cycle with a latent period of approximately 40 min and a burst period of approximately 60 min. Whole-genome sequencing confirmed the absence of virulence or antibiotic resistance genes. *In vivo*, both prophylactic and therapeutic oral administration significantly improved survival rates (100% and 90%, respectively) compared with the challenge group (30%) and reduced intestinal expression of inflammatory cytokines (IL-1, IL-6, TNF-α), restored the expression of ZO-1 and Claudin, whereas Occludin expression remained unchanged, and attenuated histopathological damage in the intestine and heart in the oral gavage model, whereas hepatic pathology remained unaffected in the oral model; however, hepatoprotective effects were observed in the supplementary intraperitoneal challenge model. 16S rRNA sequencing, transcriptomics, and metabolomics revealed that phage treatment restored gut microbiota balance (enrichment of Firmicutes, reduction of Proteobacteria/Enterobacteriaceae), modulated host energy metabolism and protein synthesis pathways, and mitigated infection-induced metabolic disturbances.

**Discussion:**

VB-SalS-XND03 exhibits potent lytic activity, environmental stability, and a favorable safety profile. Its efficacy in a physiologically relevant oral infection model, supported by integrated multi-omics analyses, highlights its potential as a promising biocontrol candidate for *Salmonella* infections in poultry.

## Introduction

*Salmonella* is a major zoonotic foodborne pathogen that poses a serious threat to public health and leads to significant economic losses in the poultry industry (Lee et al., 2025; Ssemanda et al., 2025; Sanni et al., 2023). In infected chicks, the bacterium can cross the intestinal barrier and disseminate into the bloodstream, triggering systemic inflammatory responses, septicemia, and mortality (Enz et al., 2025; Hanafy et al., 2026). In recent years, the extensive and prolonged use of antibiotics in livestock production has driven the emergence of multidrug-resistant *Salmonella* strains, therefore reducing the effectiveness of conventional antimicrobial therapies (Hong et al., 2026; Orhan et al., 2026). These challenges highlight the need to develop safe, effective, and environmentally sustainable alternative strategies for controlling *Salmonella* infections in poultry.

Bacteriophages, which are natural viruses that specifically infect bacteria, offer high host specificity, rapid self-replication, minimal disruption of commensal microbiota, and environmental compatibility. These features make them promising alternatives to antibiotics (Nie et al., 2025; Rahman et al., 2026; Sampath et al., 2025). Increasing evidence indicates that lytic bacteriophages can effectively control *Salmonella* infections in poultry through multiple mechanisms, including direct bacterial lysis, inhibition of intestinal pathogen colonization, modulation of host immune responses, and regulation of gut microbiota composition (Rahman et al., 2026; Cufaoglu et al., 2026; Zhao et al., 2025). However, currently available phage resources for *Salmonella* control remain limited. Existing phages show considerable variability in host range, environmental stability, *in vivo* safety, and their effects on the gut microbiota (Segundo-Arizmendi et al., 2025; Cufaoglu et al., 2026; Yu et al., 2026). Therefore, the isolation and characterization of novel bacteriophages with favorable biological properties and safety profiles, together with systematic evaluation of their protective efficacy and mechanisms *in vivo*, are essential for advancing the clinical application of phage therapy.

In this study, a lytic bacteriophage, designated VB-SalS-XND03, was isolated from poultry farm sewage using a clinically isolated multidrug-resistant *Salmonella enterica* serovar Enteritidis strain J3 as the host. The phage was comprehensively characterized through biological assays, whole-genome sequencing (WGS), a chick infection model, and gut microbiota analysis. These approaches were used to evaluate its host range, environmental stability, genomic safety, protective efficacy against *Salmonella* infection *in vivo*, and its modulatory effects on the gut microbiome. The findings provide a candidate phage and a theoretical foundation for developing phage-based biocontrol strategies against *Salmonella* infections in poultry.

## Materials and methods

### Bacterial strains and growth conditions

A total of 43 *Salmonella* strains (20 human and 23 animal isolates) were confirmed by PCR targeting the *invA* gene (Li et al., 2012) and cultured in RV broth at 37 °C with shaking, then stored at −80 °C in LB broth containing 25% (v/v) glycerol.

### Isolation and purification of phage

The host strain used for isolation was *S. Enteritidis* J3, a multidrug-resistant clinical isolate from diarrheic chicken feces, resistant to ampicillin, amoxicillin-clavulanic acid, ceftazidime, and streptomycin (serotype O9, ST11), and carrying resistance genes (*bla*_TEM-1_, *aph(3″)-Ib, sul2*, and *aph(6)-Id*) and virulence genes (*invA, spvB*, and *sopB*). Phage isolation was performed as described by Kazibwe et al. (2020) with minor modifications. Sewage samples from a chicken farm in Changji, China, were enriched with strain J3 in LB broth at 37 °C for 6 h. After centrifugation and filtration (0.22 μm), phages were isolated by double-layer agar (DLA) (Kropinski et al., 2009) and purified through 3–5 successive rounds.

### Phage morphology

Phage morphology was examined using transmission electron microscopy (TEM) as described previously (Ackermann, 2009). A high-titer phage suspension (10⁸ PFU/mL) was adsorbed onto a copper grid, negatively stained with 2% phosphotungstic acid, and observed under an HT7800 transmission electron microscope (Hitachi, Japan).

### Phage lytic spectrum determination

Host range was evaluated using a modified DLA method (Asghar et al., 2022). For each of the 43 *Salmonella* strains, 100 μL of bacterial culture was mixed with LB semi-solid agar and poured onto LB agar plates. Purified phage filtrate (5 μL) was spotted onto each plate. After absorption, plates were incubated overnight at 37 °C. Lytic activity was assessed by the presence of clear or turbid zones.

### Multiplicity of infection (MOI) assay

The optimal MOI was determined following a modified protocol (Li et al., 2019). Bacterial cultures were co-incubated with phage at MOIs of 100, 10, 1, 0.1, and 0.01 in LB broth at 37 °C for 6 h with shaking. Following centrifugation, the supernatant was filtered (0.22 μm), and phage titers were quantified by the DLA method. The MOI yielding the highest titer was selected as optimal.

### Phage one-step growth (OSG) curve assay

OSG kinetics were analyzed as previously described (Yu et al., 2016) with minor modifications. Phage and logarithmic-phase host bacteria were mixed at the optimal MOI and incubated at 37 °C for 10 min to allow adsorption. Following centrifugation, the pellet was washed twice, resuspended in pre-warmed LB broth, and incubated for 2 h at 37 °C with shaking. Aliquots were taken at 10-min intervals, and phage titers were determined using DLA in triplicate.

### Thermal and pH stability

Thermal stability was assessed by incubating phage at 4–80 °C for 1 h with titers measured at 20, 40, and 60 min; pH stability by incubating phage in LB broth adjusted to pH 2–13 at 37 °C for 1 h. Residual titers were determined by DLA in triplicate across three independent experiments..

### Isolation and stability determination of phage-resistant bacteria

Phage-resistant mutants were enriched by serial passage of host bacteria with phage for five cycles as described by Khan et al. (2022). Five morphologically distinct colonies were selected and further passaged five times with the original phage to confirm resistance stability.

### Phage genome sequencing and analysis

Phage genomic DNA was extracted using a commercial kit (Geneaid, Taiwan, China) and subjected to WGS on an Illumina HiSeq™ platform (Sangon Biotech, Shanghai, China). Genome assembly was performed using SPAdes 3.5.0, GapFiller 1.11, and PrInSeS-G 1.0.0. The genome was screened against VFDB and CARD for virulence and resistance genes. Annotation was performed with Prokka, and a circular genome map was generated using CGView Server. For phylogenetic analysis, BLASTn was used to retrieve the 10 most similar sequences from NCBI. A neighbor-joining phylogenetic tree was constructed in MEGA11 with 1,000 bootstrap replicates, and genome synteny was visualized using Easyfig.

### *In vivo* efficacy: Oral gavage model (Primary model)

All animal experiments were approved by the Animal Ethics Committee of Xinjiang Agricultural University (No. 2025004) and performed in accordance with ARRIVE guidelines. Three-day-old healthy chicks obtained from a commercial hatchery were housed on sterile bedding with *ad libitum* access to feed and water and randomly assigned to five groups. To reduce colonization resistance and facilitate consistent intestinal infection, all chicks received streptomycin (25 mg/kg) by oral gavage one day before *Salmonella* challenge. On the day of infection, chicks were fasted for 12 h, with water withheld during the final 4 h before gavage to minimize gastric contents and ensure consistent dosing. Two independent oral gavage experiments were performed: Experiment 1 for survival analysis (n = 8 per group) and Experiment 2 for mechanistic investigations (n = 20 per group).

The challenge group (S) received 500 µL of *Salmonella* J3 suspension (5.34 × 10⁹ CFU/mL) by oral gavage at 0 h, followed by 500 µL of sterile PBS. The treatment group (Z) received 500 µL of *Salmonella* J3 at 0 h, followed immediately by 500 µL of phage suspension (1 × 10¹⁰ PFU/mL). The prevention group (Y) received 500 µL of phage suspension 12 h before bacterial challenge and was then challenged with *Salmonella* J3 at 0 h. The safety group (P) received the phage suspension alone using the same dose and route of administration. The control group (Ctrl) received sterile PBS at both time points.

In Experiment 1, mortality was recorded daily for 7 days to generate survival curves. In Experiment 2, six chicks from each group were randomly selected and euthanized at 48 h post-infection. Samples of the heart, liver, spleen, and intestinal segments (duodenum, jejunum, ileum, cecum, and rectum) were collected for histopathological examination using hematoxylin and eosin (H&E) staining. Duodenal tissues were collected for qPCR analysis of inflammatory cytokines (IL-1, IL-6, and TNF-α) and tight junction proteins (ZO-1, Claudin, and Occludin), and additional duodenal samples were snap-frozen for transcriptomic analysis. Cecal contents were collected for 16S rRNA sequencing (n = 4 per group), and blood samples were obtained from the jugular vein for serum metabolomic analysis. All histopathological and molecular analyses were performed by investigators blinded to the treatment groups.

### Supplementary *in vivo* model: Intraperitoneal injection

To further validate the therapeutic efficacy of VB-SalS-XND03 against severe systemic infection, an additional intraperitoneal (i.p.) challenge model was performed as supplementary validation. Briefly, chicks (n = 10 per group) received an i.p. injection of 200 µL *Salmonella* J3 suspension (5.34 × 10⁸ CFU/mL). Immediately after bacterial challenge, 200 µL of phage suspension (1 × 10¹⁰ PFU/mL) was administered by oral gavage. A lower bacterial inoculum was used in the i.p.model because systemic administration bypasses the intestinal barrier, whereas a higher oral dose is required to achieve reliable intestinal colonization. Survival was monitored daily for 7 days. At the experimental endpoint, serum TNF-α concentrations and histopathological changes in the liver, spleen, and heart were evaluated in a blinded manner.

### 16S rRNA sequencing and bioinformatics

Cecal DNA was extracted, and the V3–V4 region of the 16S rRNA gene was amplified using primers 515F/806R (Caporaso et al., 2011) and sequenced on an Illumina NovaSeq 6000 platform (PE250). Sequence data were processed using QIIME2 (Bolyen et al., 2019). Beta-diversity analyses were performed using PCoA and UPGMA. Linear discriminant analysis effect size (LEfSe) was performed to identify differentially abundant taxa.

### Transcriptomics and metabolomics

Total RNA was extracted from duodenal tissues and subjected to transcriptome sequencing by Novogene (Beijing, China). Serum samples were analyzed using untargeted metabolomics (LC-MS/MS). Differential expression analysis (|log₂FC| ≥ 0.58, padj ≤ 0.05) and differential metabolites (VIP ≥ 1, *P* ≤ 0.05) were identified. Gene Ontology (GO) and Kyoto Encyclopedia of Genes and Genomes (KEGG) pathway enrichment analyses were then performed.

### Statistical analysis

Data are presented as the mean ± SD. Comparisons among multiple groups were performed using one-way ANOVA followed by Tukey's post hoc test in GraphPad Prism version 9.0. Survival curves were analyzed using the Kaplan–Meier method and compared using the log-rank test. A *P* < 0.05 was considered statistically significant.

## Results

### Isolation, purification, morphology, and biological characteristics of phage VB-SalS-XND03

**Fig. 1:**
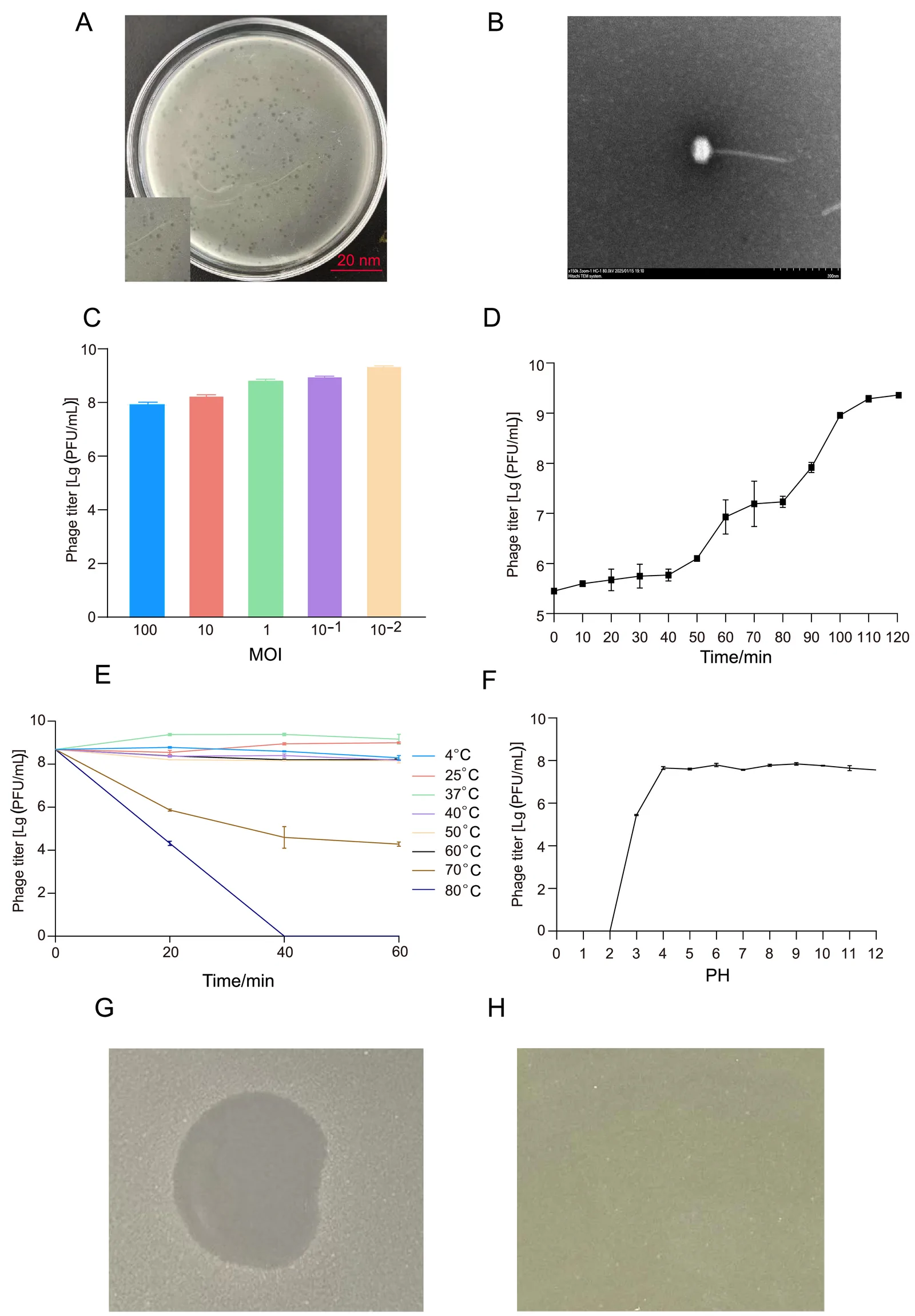
**Biological characteristics of phage VB-SalS-XND03.** (A) Plaque morphology. (B) Transmission electron micrograph (scale bar: 100 nm). (C) Optimal MOI; (D) One-step growth curve. (E) Effect of temperature on stability. (F) Effect of pH on phage stability. (G) Host bacterium and phage. (H) Resistant strain and phage.

Using *S. Enteritidis* J3 as the host strain, a bacteriophage, VB-SalS-XND03, was isolated from sewage samples. Following purification, the phage produced clear, round plaques of uniform size (Fig. 1A). TEM showed an icosahedral head structure (Fig. 1B). Host range analysis revealed that VB-SalS-XND03 lysed 10 of 43 *Salmonella* strains, including nine *S. Enteritidis* strains, which produced clear lytic zones, and one *S. Potsdam* strain, which produced a weak turbid zone, indicating preferential lytic activity against *S. Enteritidis* (Table 1). The optimal MOI was 0.01 (Fig. 1C). The OSG curve showed a latent period of ∼40 min and a burst period of ∼60 min, reaching a final titer >10⁹ PFU/mL (Fig. 1D). Stability assays showed the phage remained stable at temperatures 4–60 °C and pH 4–12, with titer reduction at 70 °C and complete inactivation at 80 °C (Fig. 1E–F). All five independently isolated phage-resistant bacterial mutants retained stable resistance after five serial passages, indicating that the resistance phenotype was maintained (Fig. 1G–H).

**Table 1:** The lytic spectrum of phage VB-SalS-XND03.

Isolate	Serotype	Source	MLST typing	Antigen	Droplet results
H8	*S. Enteritidis*	Human	ST34	9;g,m;-	++
H10	*S. Enteritidis*	Human	ST11	9;g,m;-	+++
H16	*S. Enteritidis*	Human	ST11	9;g,m;-	+++
H17	*S. Enteritidis*	Human	ST11	9;g,m;-	++
H18	*S. Enteritidis*	Human	ST11	7;g,m;-	+++
H22	*S. Enteritidis*	Human	ST11	9;g,m;-	+++
J1	*S. Potsdam*	Human	ST2039	7;l,v;e,n,z15	+
J3	*S. Enteritidis*	Chicken	ST11	9;g,m;-	+++
J4	*S. Enteritidis*	Chicken	ST11	9;g,m;-	+++
M3	*S. Enteritidis*	Horse	ST251	4;a;e,n,x	+++

Note: +++, strong lysis (clear zone); ++, moderate lysis; +, weak lysis (turbid zone).

### Genomic and phylogenetic analysis

**Fig. 2:**
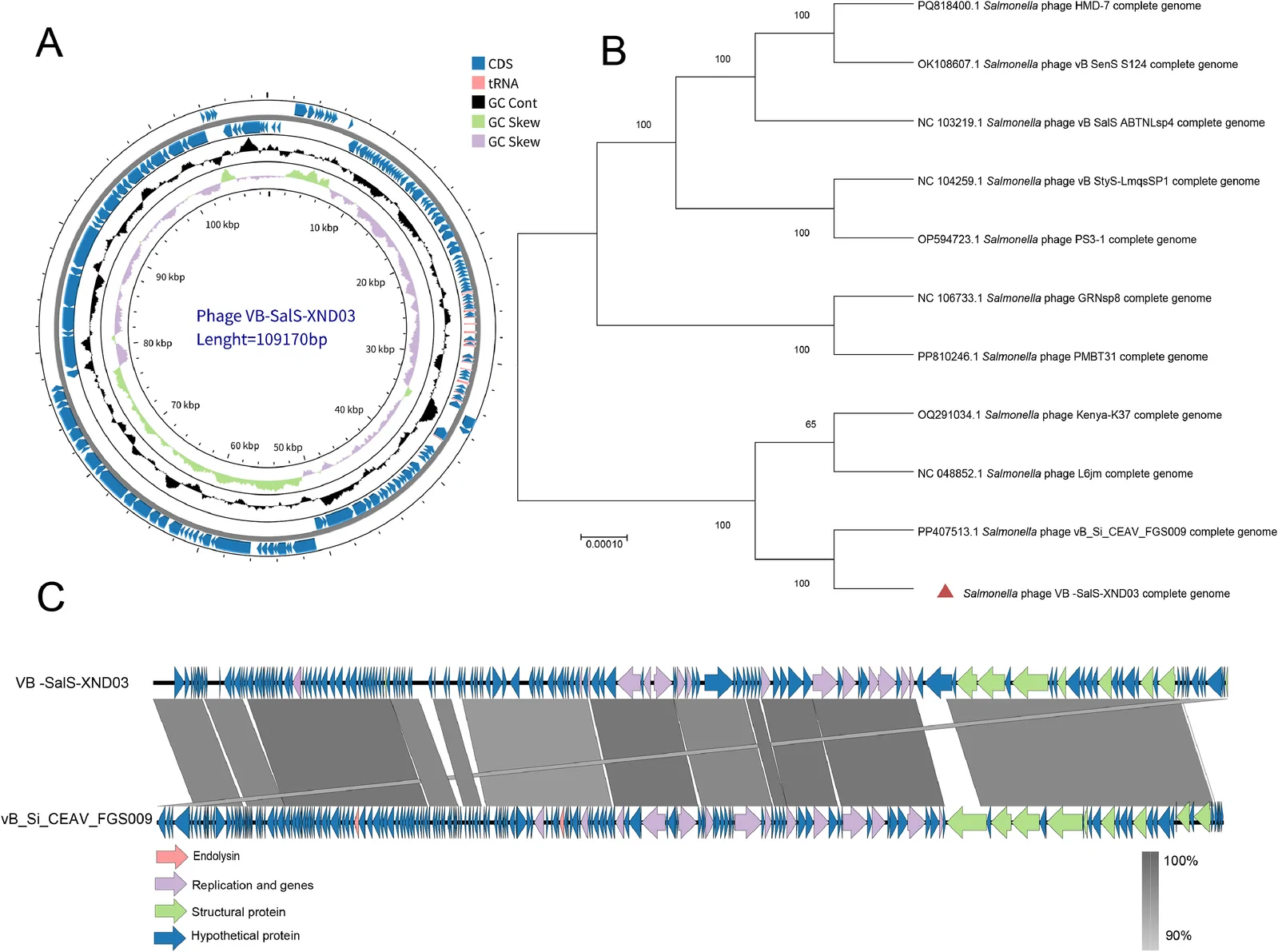
**Genomic characterization.** (A) Circular genome map. (B) Phylogenetic tree. (C) Whole-genome synteny analysis with vB_Si_CEAV_FGS009.

WGS revealed a genome size of 109,170 bp with a GC content of 38.99%, encoding 161 ORFs (Fig. 2A). No virulence factors, antibiotic resistance genes, or lysogeny-related genes were identified. The sequence was deposited in NCBI (PZ049449). Phylogenetic analysis showed the highest similarity to phage vB_Si_CEAV_FGS009, clustering within the same branch (Fig. 2B). Synteny analysis confirmed moderate genomic similarity (>76%) (Fig. 2C). Based on these features, VB-SalS-XND03 was classified into the genus *Tequintavirus*.

### Protective efficacy of oral phage administration against oral *Salmonella* challenge (Primary model)

**Fig. 3:**
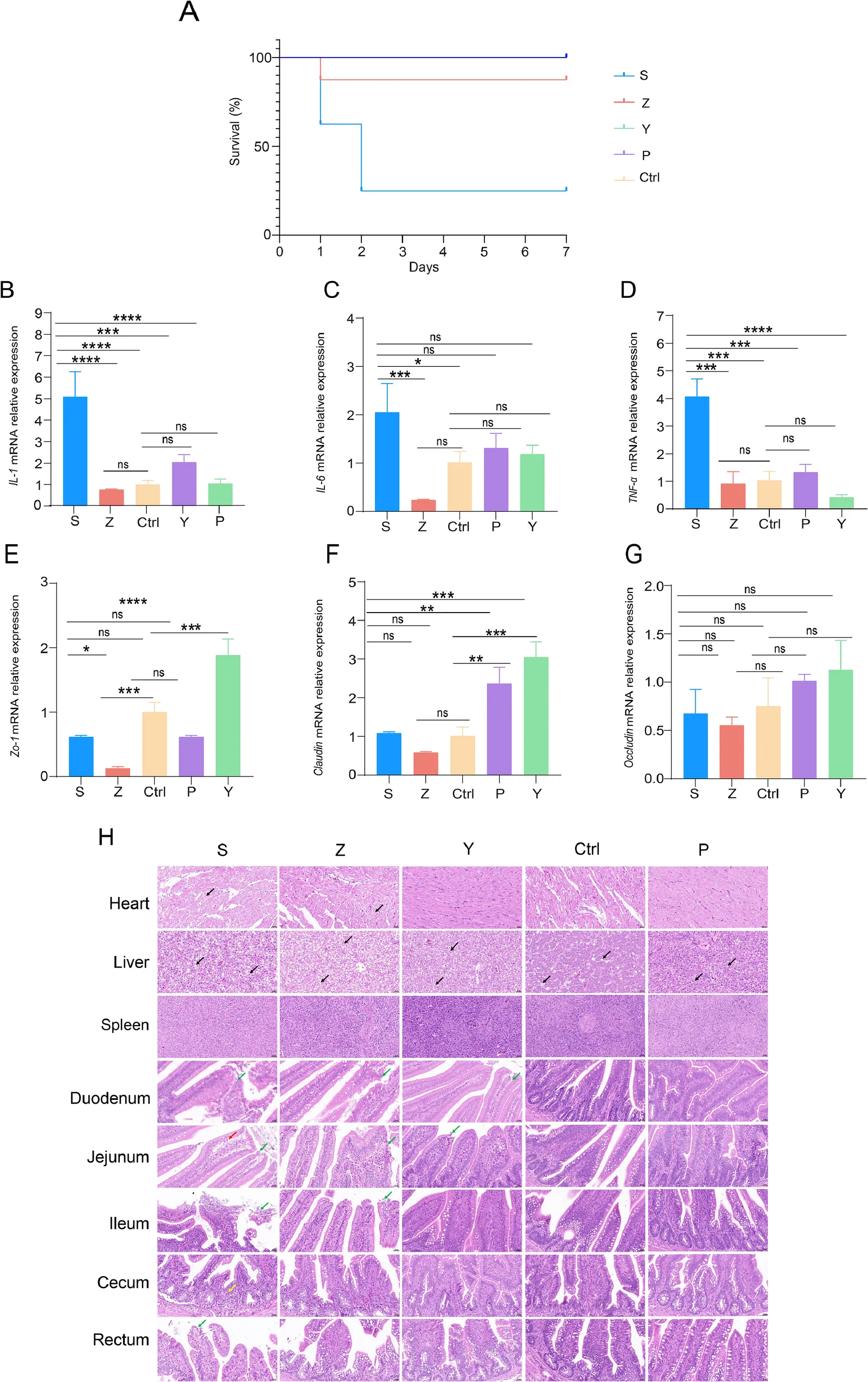
**Protective efficacy of oral phage administration against oral *Salmonella* challenge.** (A) Survival curves (n=8). (B–D) Relative mRNA expression of IL-1, IL-6, TNF-α in duodenum. (E–G) Relative mRNA expression of ZO-1, Claudin, Occludin. (H) Histopathological examination of heart and intestinal segments (H&E, ×400). Heart: myocardial edema; Liver: hepatocellular degeneration; Intestine: epithelial shedding, villous separation, and inflammatory infiltration (arrows indicate representative lesions).

VB-SalS-XND03 administered by oral gavage exhibited significant protective efficacy (Fig. 3). The survival rate in the challenge group (S) was 30%, whereas it increased to 90% in the treatment group (Z) and 100% in the prevention group (Y). The Ctrl and P groups showed 100% survival (Fig. 3A). qPCR analysis of duodenal tissues showed that the mRNA expression levels of the inflammatory cytokines IL-1, IL-6, and TNF-α were significantly increased in the S group (*P* < 0.01). Expression levels in the Z and Y groups were significantly reduced and were comparable to those of the Ctrl group (Fig. 3B–D). The mRNA expression levels of the tight junction proteins ZO-1 and Claudin were significantly reduced in the S group (*P* < 0.05), whereas Occludin expression did not differ significantly among the groups. Oral phage administration restored ZO-1 and Claudin expression to levels approaching those observed in the Ctrl group (Fig. 3E–G).

Histopathological examination (Fig. 3H) revealed that *Salmonella* challenge caused myocardial edema characterized by cellular swelling and pale, loose cytoplasm, as well as extensive intestinal lesions including epithelial shedding in the duodenum and cecum, separation of the jejunal epithelium from the lamina propria, and edema of ileal villi. These pathological alterations were markedly alleviated in the Z and Y groups, with reduced myocardial edema and improved intestinal architecture. In the liver, the challenge group exhibited widespread severe hepatocellular degeneration. Similar degrees of degeneration were observed in both the Z and Y groups, indicating that phage administration did not result in a significant improvement in hepatic pathology during the experimental period. Only mild hepatocellular degeneration was observed in the Ctrl and P groups (Fig. 3H). No significant pathological changes were observed in the safety evaluation or control groups, confirming the safety of phage VB-SalS-XND03 and its protective effect against *Salmonella*-induced intestinal and myocardial damage, whereas hepatic pathology remained largely unaffected in this oral challenge model.

### 16S rRNA sequencing analysis of gut microbiota

**Fig. 4:**
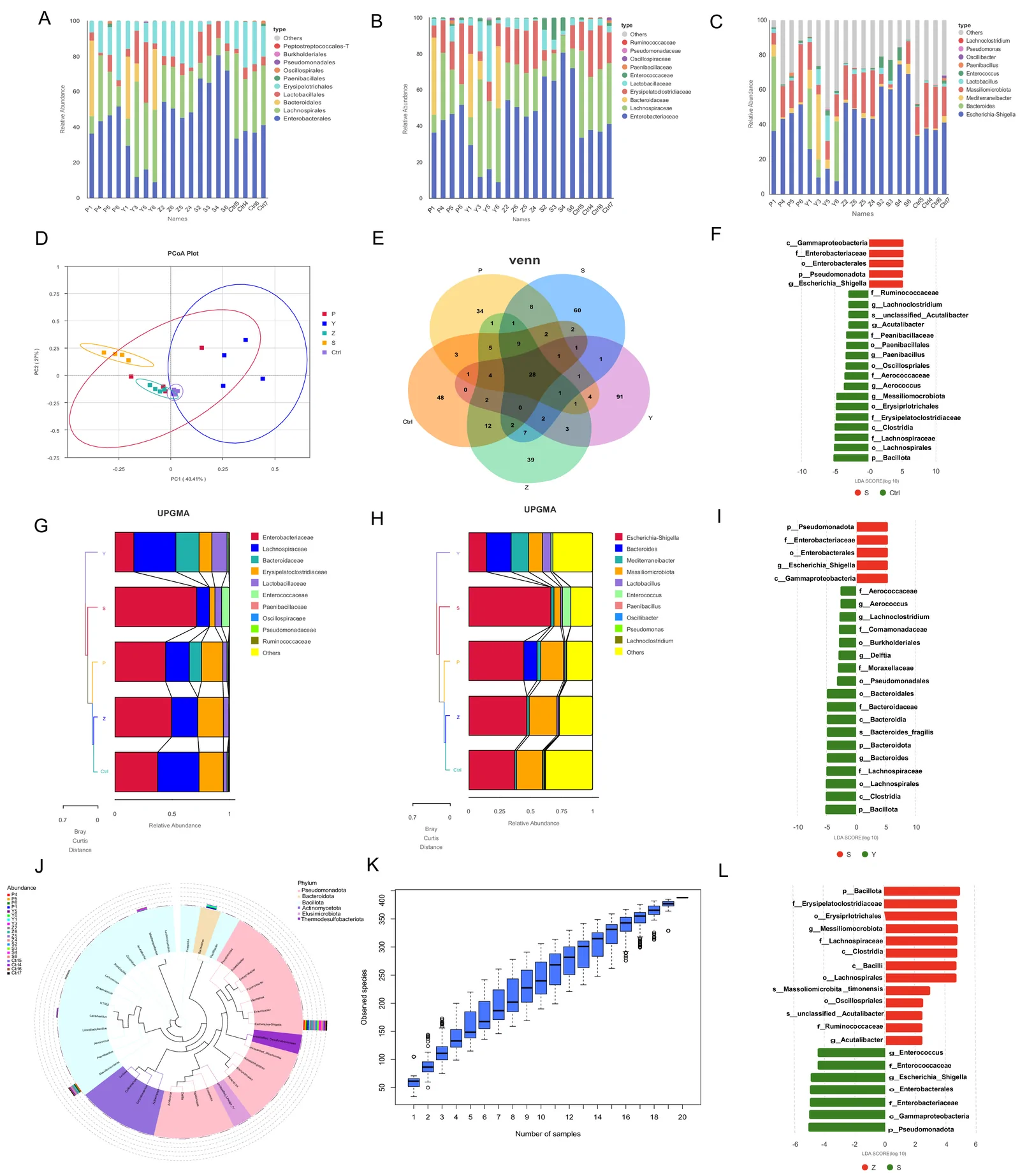
**16S rRNA sequencing analysis of gut microbiota (n** = **4/group)**. (A–C) Relative abundance of the gut microbiota at the family, order, and genus levels. (D) PCoA based on weighted UniFrac distances. (E) Venn diagram of OTUs. (F) LEfSe analysis (S vs Ctrl). (G) UPGMA clustering at the family level. (H) UPGMA clustering at the genus level. (I) LEfSe analysis (S vs Y). (J) Phylogenetic tree of dominant bacterial taxa. (K) Species accumulation curve. (L) LEfSe analysis (S vs Z).

To evaluate the effects of the gut microbiota, 16S rRNA sequencing was performed (n = 4 per group). Venn diagram analysis identified a shared core microbiota among all groups together with group-specific taxa (Fig. 4E). The phylogenetic tree further illustrated the phylogenetic relationships and relative abundances of the dominant bacterial taxa across the experimental groups (Fig. 4J). Beta-diversity analyses using PCoA and UPGMA demonstrated that the Z and Y groups clustered closely with the Ctrl group, whereas the S group formed a distinct cluster, indicating that phage intervention partially restored the gut microbial community structure (Fig. 4D, G, H).

Taxonomic composition analysis showed that *Salmonella* infection increased the relative abundance of Proteobacteria (Pseudomonadota), Enterobacteriaceae, and *Escherichia–Shigella*, while reducing the abundance of beneficial taxa, including Lachnospiraceae, Ruminococcaceae, and *Lactobacillus* (Fig. 4A–C). Phage administration partially reversed these changes by reducing opportunistic pathogens and restoring beneficial bacterial taxa. LEfSe further confirmed these microbial shifts (Fig. 4F, I, L). The species accumulation curve reached a plateau, indicating adequate sequencing depth (Fig. 4K).

### Transcriptomic and metabolomic profiling reveal systemic host modulation

**Fig. 5:**
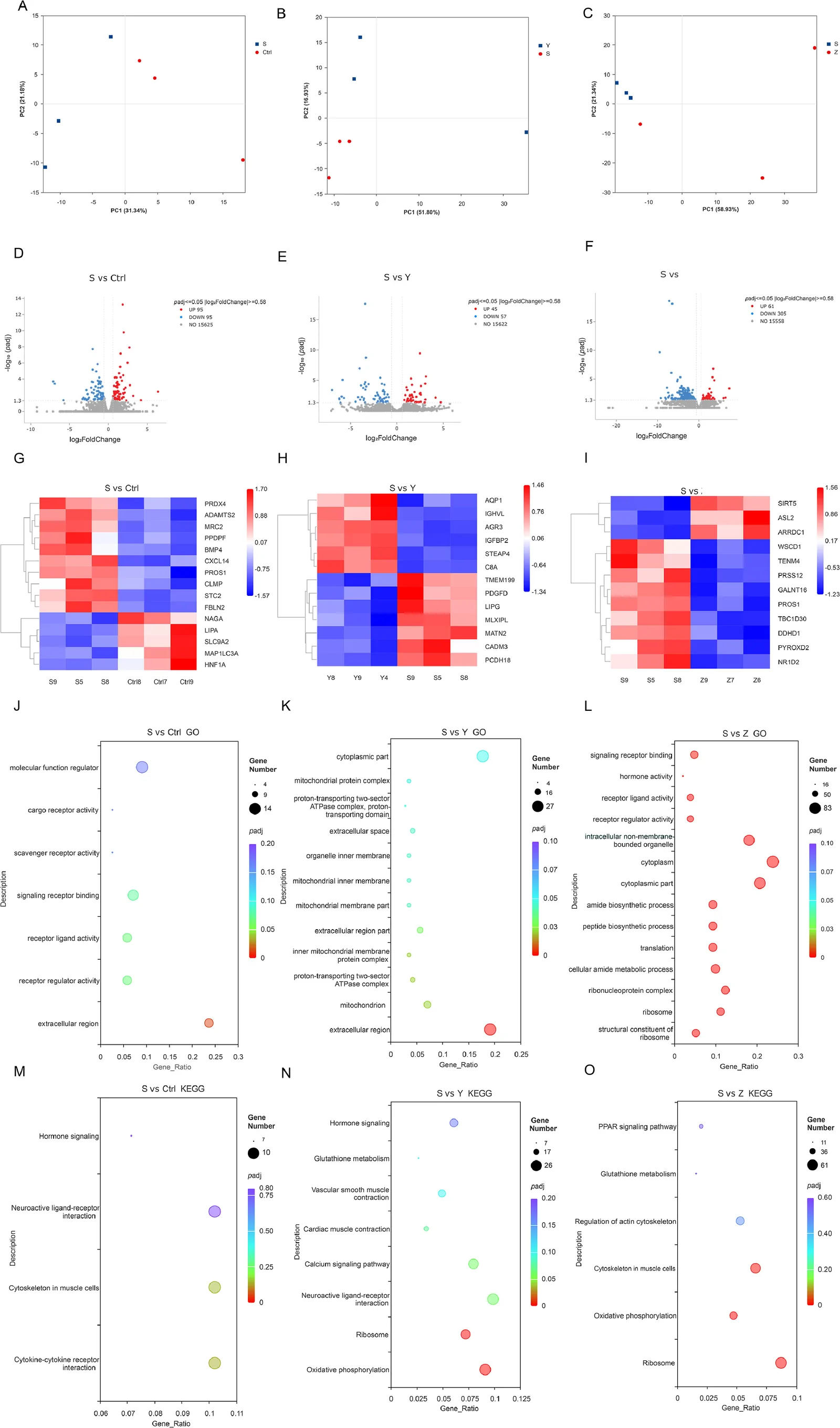
**Transcriptomic profiling**. (A–C) PCA of S vs Ctrl, S vs Y, S vs Z. (D–F) Volcano plots of DEGs. (G–I) Heatmaps of DEGs. (J–O) GO and KEGG enrichment analyses for S vs Ctrl, S vs Y, and S vs Z.

PCA of the transcriptomic data demonstrated clear separation of the S group from the Ctrl, Y, and Z groups, indicating marked infection-induced transcriptional changes that were partially restored following phage administration (Fig. 5A–C). Differential expression analysis identified hundreds of DEGs in the comparisons of S versus Ctrl, S versus Y, and S versus Z (Fig. 5D–F). Hierarchical clustering further demonstrated distinct gene expression profiles among the experimental groups (Fig. 5G–I). Compared with the Ctrl group, the S group exhibited extensive transcriptional changes, whereas the transcriptomic profiles of the Z and Y groups shifted toward those of the Ctrl group, indicating partial recovery of infection-associated gene expression patterns following phage intervention. GO and KEGG pathway enrichment analyses showed that prophylactic phage administration (Y) primarily affected pathways associated with mitochondrial function and oxidative phosphorylation, whereas therapeutic phage administration (Z) predominantly regulated ribosome-associated processes, protein translation, and cytoskeletal remodeling (Fig. 5J–O).

**Fig. 6:**
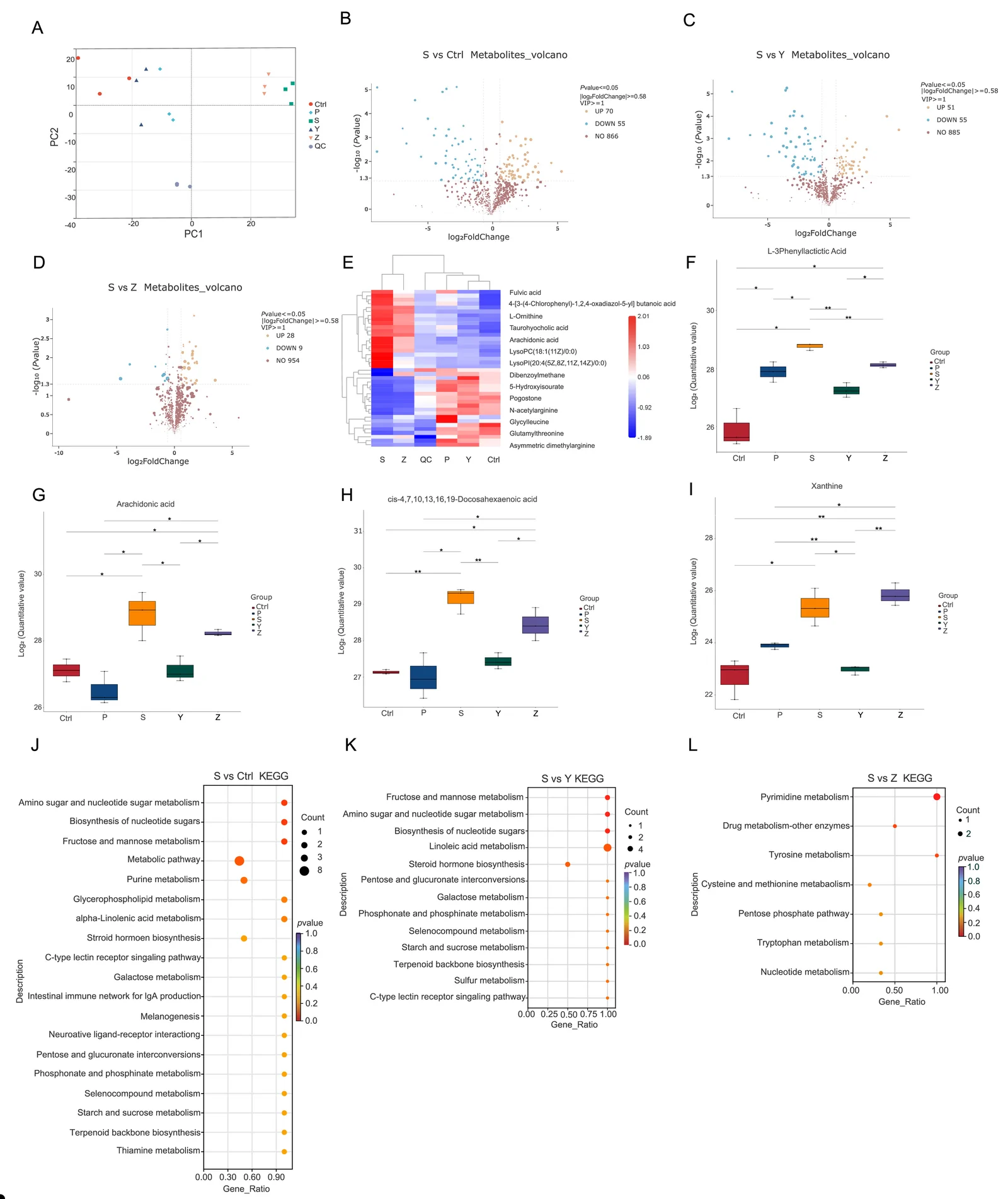
**Untargeted metabolomic profiling.** (A) PCA score plot. (B–D) Volcano plots of differential metabolites. (E) Hierarchical clustering heatmap. (F–I) Relative abundance of representative metabolites (L-3-phenyllactic acid, arachidonic acid, DHA, xanthine). (J–L) KEGG pathway enrichment analyses.

Untargeted metabolomic analysis demonstrated distinct metabolic profiles among the experimental groups (Fig. 6A). The S group exhibited metabolic alterations, many of which were partially restored following phage administration. Differential metabolite analysis identified pronounced metabolic remodeling in both the Y and Z groups, whereas only limited metabolic changes were observed in the P group (Fig. 6B–E). Representative metabolites, including arachidonic acid and L-3-phenyllactic acid, showed trends toward normalization following phage treatment. KEGG pathway analysis indicated that the altered metabolites were primarily associated with carbohydrate, lipid, nucleotide, and amino acid metabolism (Fig. 6F–L).

### Supplementary validation using the intraperitoneal challenge model

To further validate the protective efficacy against severe systemic infection, an additional intraperitoneal challenge model was established. In contrast to the oral gavage model, in which hepatic pathology was not improved, the intraperitoneal challenge model revealed that oral phage administration significantly increased the survival rate (90% in the treatment group versus 30% in the challenge group), reduced serum TNF-α concentrations, and alleviated histopathological damage in the liver, spleen, and heart, in addition to the intestines (Supplementary Fig. S1), further supporting the protective efficacy of VB-SalS-XND03 against systemic *Salmonella* infection.

## Discussion

Phage therapy has gained increasing attention as a promising alternative to control multidrug-resistant *Salmonella* infections in poultry (Wanasawaeng et al., 2025; Shahdadi et al., 2024). In this study, we isolated VB-SalS-XND03 from poultry sewage and systematically characterized its biological properties, genomic safety, and therapeutic efficacy. By employing an oral gavage model that closely mimics the natural fecal-oral transmission route and integrating multi-omics analyses, we further investigated the mechanisms underlying its protective effects.

VB-SalS-XND03 exhibited serotype-preferential lytic activity, effectively lysing all 9 of 10 tested *S. Enteritidis* strains while showing reduced activity against the tested *S. Typhimurium*. Compared with the broad-spectrum phage vB_SalP_LDW16 (lysis rate 88%) reported by Cao et al. (2022), VB-SalS-XND03 has a relatively narrow host range. Although narrow-host-range phages require accurate pathogen identification before application, their specificity offers a key advantage: precise targeting of pathogenic bacteria while preserving the beneficial commensal microbiota (Chen et al., 2025). This characteristic may be particularly valuable for targeted control of *S. Enteritidis* and could support its incorporation into phage cocktail formulations in future field applications (Olawade et al., 2024).

The growth characteristics and environmental stability of VB-SalS-XND03 further support its potential practical application. The OSG curve showed a latent period of approximately 40 min and a burst period of 60 min, with a final titer exceeding 10⁹ PFU/mL. This replication cycle is faster than that of SEpBS-1, which exhibited a latent period of approximately 2 h and a burst period of 2–3.5 h (Wanasawaeng et al., 2025). Such rapid proliferation may enable early suppression of pathogen multiplication at the infection site, improving therapeutic outcomes. Furthermore, VB-SalS-XND03 remained stable over a broad pH range (4–12) and at temperatures up to 60 °C, indicating strong environmental tolerance. This stability exceeds that reported for some previously described *Salmonella* phages that remain stable only within a pH range of 5–9 (Wanasawaeng et al., 2025). However, phage activity decreased significantly at 70 °C and was completely inactivated at 80 °C, suggesting that high-temperature feed processing procedures, such as pelleting, may compromise phage viability. Therefore, post-pelleting supplementation or alternative delivery approaches may be required for practical field applications.

Genomic safety is an essential criterion for the development of therapeutic phages. Whole-genome sequencing showed that VB-SalS-XND03 possesses a 109,170 bp genome with a GC content of 38.99% and contains 161 predicted open reading frames. Importantly, no lysogeny-associated genes, virulence factors, or antimicrobial resistance genes were identified. The strictly lytic lifestyle and the absence of potentially transmissible, undesirable genetic elements satisfy key safety requirements for therapeutic phages, as highlighted by Shaji et al. (2023). Phylogenetic and genome synteny analyses indicated a close relationship with phage vB_Si_CEAV_FGS009, with more than 76% genomic similarity and conserved genome organization, suggesting a possible common evolutionary origin. These genomic features provide a solid safety basis for its subsequent evaluation in animal infection models.

The protective efficacy of VB-SalS-XND03 was evaluated using a physiologically relevant oral gavage model. Both prophylactic and therapeutic phage administration significantly improved survival, reaching 100% and 90%, respectively, compared with 30% in the challenge group. The slightly better protection observed with prophylactic administration is likely due to the presence of phage at the intestinal mucosal surface before bacterial exposure, enabling earlier suppression of bacterial proliferation and limiting subsequent tissue injury and inflammatory responses. This observation is consistent with previous studies demonstrating that early or prophylactic phage administration provides better protection against *Salmonella* infection in chickens (Li et al., 2025; Zheng et al., 2024). At the molecular level, phage treatment significantly reduced the duodenal mRNA expression of the pro-inflammatory cytokines IL-1, IL-6, and TNF-α while restoring the expression of the tight junction proteins ZO-1 and Claudin. Occludin expression was not significantly affected. These findings suggest that VB-SalS-XND03 protects intestinal barrier integrity while limiting excessive inflammatory responses, both of which are important for reducing *Salmonella*-induced intestinal injury (Ijaz et al., 2021).

Histopathological analysis showed mild hepatocellular degeneration in both the control and safety groups, characterized by pale cytoplasm and occasional small cytoplasmic vacuoles with preserved hepatic sinusoidal architecture. This phenomenon may be associated with the short-term fasting and water restriction protocol implemented before oral gavage, which has been reported to induce transient hepatic glycogen depletion and similar morphological changes (Zhang et al., 2019). However, the challenge, treatment, and prevention groups exhibited significantly more extensive and severe hepatocellular degeneration (Fig. 3H). Although the precise causes of both the background and infection-associated hepatic lesions require further investigation, the significantly higher severity observed following *Salmonella* challenge suggests that these pathological changes were primarily associated with systemic infection rather than the fasting procedure alone. However, the fact that phage administration did not alleviate this hepatic damage suggests that oral phage delivery, while effective in the intestine and heart, was insufficient to protect the liver under these conditions—a finding that contrasts with the hepatoprotective effect observed in the supplementary intraperitoneal challenge model.

Interestingly, the hepatoprotective effect was evident only in the intraperitoneal challenge model, not in the oral gavage model. This discrepancy may reflect route-dependent differences in phage bioavailability: after oral administration, phage particles must traverse the gastrointestinal tract and may be diluted or partially inactivated before reaching the portal circulation, whereas in the i.p. model, the systemic route may allow more direct delivery to the liver. Alternatively, the severe hepatocellular degeneration observed in the oral model may be driven by bacterial translocation over a prolonged period (48 h), during which irreversible damage had already occurred, whereas the i.p. model, with a lower inoculum and more acute systemic challenge, may permit phage to exert a protective effect before extensive hepatic injury develops. These possibilities warrant future pharmacokinetic and time-course studies.

The impact of phage treatment on gut microbiota homeostasis was further assessed by 16S rRNA sequencing. *Salmonella* infection caused pronounced gut microbial dysbiosis, characterized by increased abundances of Proteobacteria (Pseudomonadota), Enterobacteriaceae, and *Escherichia–Shigella*, accompanied by reduced abundances of beneficial taxa, including Lachnospiraceae, Ruminococcaceae, *Lactobacillus*, and Massiliomicrobiota. These results are consistent with previous reports showing that *Salmonella* infection promotes the expansion of Enterobacteriaceae and disrupts gut microbial homeostasis in chickens (Mon et al., 2015; Khan et al., 2020). Both prophylactic and therapeutic phage administration partially restored the microbial community toward that of the healthy control group by reducing the abundance of opportunistic pathogens while increasing beneficial bacterial taxa. Moreover, the gut microbiota of the P group remained highly similar to that of the Ctrl group, indicating that VB-SalS-XND03 caused minimal disturbance to the native intestinal microbiota. This selective antibacterial activity is a key advantage of phage therapy over broad-spectrum antimicrobials and supports the potential application of VB-SalS-XND03 as a targeted biocontrol agent.

To further elucidate the host molecular responses following phage treatment, we performed transcriptomic and metabolomic analyses. The integrated multi-omics analyses provided further mechanistic insights into the host response to phage treatment. Transcriptomic profiling revealed distinct molecular responses to prophylactic and therapeutic administration. Prophylactic treatment (Y) was primarily associated with enhanced mitochondrial function and oxidative phosphorylation pathways, whereas therapeutic treatment (Z) predominantly affected ribosome-associated protein synthesis and cytoskeletal remodeling pathways. These findings suggest that phage administration before bacterial challenge may help maintain cellular energy metabolism, whereas treatment after infection may preferentially support tissue repair and recovery. Metabolomic analysis further showed that *Salmonella* infection disrupted multiple metabolic pathways, particularly those involved in carbohydrate metabolism, including fructose and mannose metabolism, amino sugar and nucleotide sugar metabolism, and biosynthesis of nucleotide sugars. These pathways are closely related to intestinal mucus production, epithelial glycosylation, and host–microbiota interactions (Corfield, 2018; Kudelka et al., 2020). Phage administration, particularly under the prophylactic regimen, partially restored these metabolic alterations toward the profile observed in healthy controls. In the therapeutic group, differential metabolites were primarily enriched in pyrimidine metabolism, whereas amino acid metabolism and redox-related pathways showed enrichment trends. These findings suggest that post-infection treatment may preferentially regulate nucleotide metabolism and processes associated with tissue repair. These microbiota, metabolomic, and transcriptomic findings suggest that VB-SalS-XND03 protects chicks through a coordinated phage-gut microbiota-metabolism-transcriptome-barrier repair axis.

Several limitations of this study should be acknowledged. First, the 7-day observation period was sufficient to evaluate the acute therapeutic efficacy of phage treatment because mortality occurred predominantly within the first 5 days after challenge. However, this study did not assess the long-term effects of phage administration on production performance, including feed conversion ratio, body weight gain, and final market weight. Future studies with longer follow-up periods are warranted to determine whether phage treatment provides sustained benefits throughout the broiler production cycle. Second, although phage-resistant mutants were successfully isolated *in vitro* and their resistance phenotype remained stable after serial passage, the frequency, evolutionary dynamics, and biological consequences of resistance development during *in vivo* treatment were not investigated. Therefore, the potential effect of phage resistance on the long-term therapeutic efficacy of VB-SalS-XND03 remains unclear and should be addressed in future studies. Third, direct enumeration of *Salmonella* CFUs in tissue samples was not achieved because of severe interference from tissue debris, background overgrowth, and inconsistent colony recovery despite repeated optimization attempts. However, the significant reduction in Enterobacteriaceae abundance, the bacterial family that includes *Salmonella*, as determined by 16S rRNA sequencing, together with improvements in survival and histopathological findings, provides indirect evidence of reduced bacterial burden following phage treatment. Future investigations should optimize bacterial quantification methods, such as improved tissue processing protocols or qPCR-based approaches, to obtain direct measurements of bacterial load. Finally, the pharmacokinetic properties of VB-SalS-XND03, including its absorption, tissue distribution, persistence, and clearance, were not evaluated. Characterization of these parameters will be important for optimizing dosing strategies, including dose selection, administration frequency, and treatment timing, to maximize therapeutic efficacy in future applications.

Despite these limitations, this study comprehensively demonstrates that VB-SalS-XND03 possesses favorable biological characteristics, a short latent period, a rapid lytic cycle, and strong environmental stability, along with a safe genomic profile lacking virulence and antibiotic resistance genes. Its significant protective efficacy against *Salmonella* infection in a physiologically relevant oral gavage chick model is supported by improved survival, reduced inflammatory responses, restored intestinal barrier function, rebalanced gut microbiota, and modulation of host metabolic and transcriptional pathways. These findings support the potential of VB-SalS-XND03 as a promising candidate for the development of phage-based biocontrol strategies against *Salmonella* infections in poultry. Future studies should evaluate its efficacy under commercial production conditions and combine pharmacokinetic characterization with mechanistic investigations to optimize dosing strategies and facilitate practical application in poultry production.

## Conclusion

VB-SalS-XND03 possesses favorable biological characteristics, including a short latent period, a rapid lytic cycle, and broad environmental stability, together with a safe genomic profile lacking virulence and antibiotic resistance genes. In a physiologically relevant oral gavage chick model, oral administration of VB-SalS-XND03 significantly protected against *Salmonella* infection by improving survival, reducing intestinal inflammatory responses, preserving intestinal barrier integrity, and partially restoring gut microbial homeostasis. Integrated multi-omics analyses further demonstrated coordinated changes in host transcriptional programs and metabolic pathways associated with phage treatment. These results highlight the potential of VB-SalS-XND03 as a promising candidate for phage-based biocontrol of *Salmonella* infections in poultry.

## Ethics statement

All animal procedures were conducted following the guidelines approved by the Animal Ethics Committee of Xinjiang Agricultural University (No. 2025004).

## Funding

Gongliu County Science and Technology Innovation Project (GXC202507); Technology Action Project for Rural Revitalization Industry Development in Xinjiang Uygur Autonomous Region (2024NC034); Autonomous Region Science and Technology Commissioner Rural Science and Technology Entrepreneurship Action Project: Gongliu County Experimental Demonstration Station for the Construction and Industrialization of a Green Prevention and Control System Against Bacterial Diseases in Ili Chickens Based on a Combined Phage–Probiotic Approach.

Supplementary Fig. S1 **Protective efficacy in the intraperitoneal challenge model.** (A) Survival curves. (B) Serum TNF-α levels. (C) Histopathology of liver, spleen, heart, and intestines (H&E, ×200). Liver: fatty degeneration and congestion; Spleen: focal necrosis; Heart: epicardial hyperplasia and lymphocytic infiltration; Intestine: epithelial shedding, villous separation, edema, and luminal debris (arrows indicate representative lesions).

## Author contributions

Qirui Zang designed and performed the main experiments, analyzed the data, and wrote the original draft. Mingshuai Chen, Yingchao Li, Chenyang Shi, and Yaolong Song contributed to experimental procedures, data collection, and data interpretation. Ying Huang, Xueqin Lan, Wanjing Jin, Yixin Bai, Gulifeire Yilizhati, Yishan Sun, Shuhui Yang, Yan Wang, Yaqi Huang, Yuan Tian, and Wenxing Jiang participated in data analysis and interpretation of the results. Xiaofeng Zheng, Jinxin Xie, and Mengfei Zhang provided critical guidance throughout the study and jointly conceived the project with Panpan Tong. Panpan Tong supervised the overall project. All authors have read and approved the final manuscript.

## Disclosures

The authors declare no conflict of interest.

## Appendix Supplementary materials

Image, application 1

## Footnotes

Supplementary material associated with this article can be found, in the online version, at doi:10.1016/j.psj.2026.107489.
