# Supplementary material for: Isolation and identification of a chicken-derived Salmonella phage VB-SalS-XND03, Its biological characteristics and application effect against Salmonella infection in chicks
Source: Poult Sci. 2026 Jul 25;105(11):107489. doi: 10.1016/j.psj.2026.107489 (PMC13507854; doi:10.1016/j.psj.2026.107489)
Supplement: Supplementary file 1 [file mmc1.pdf]

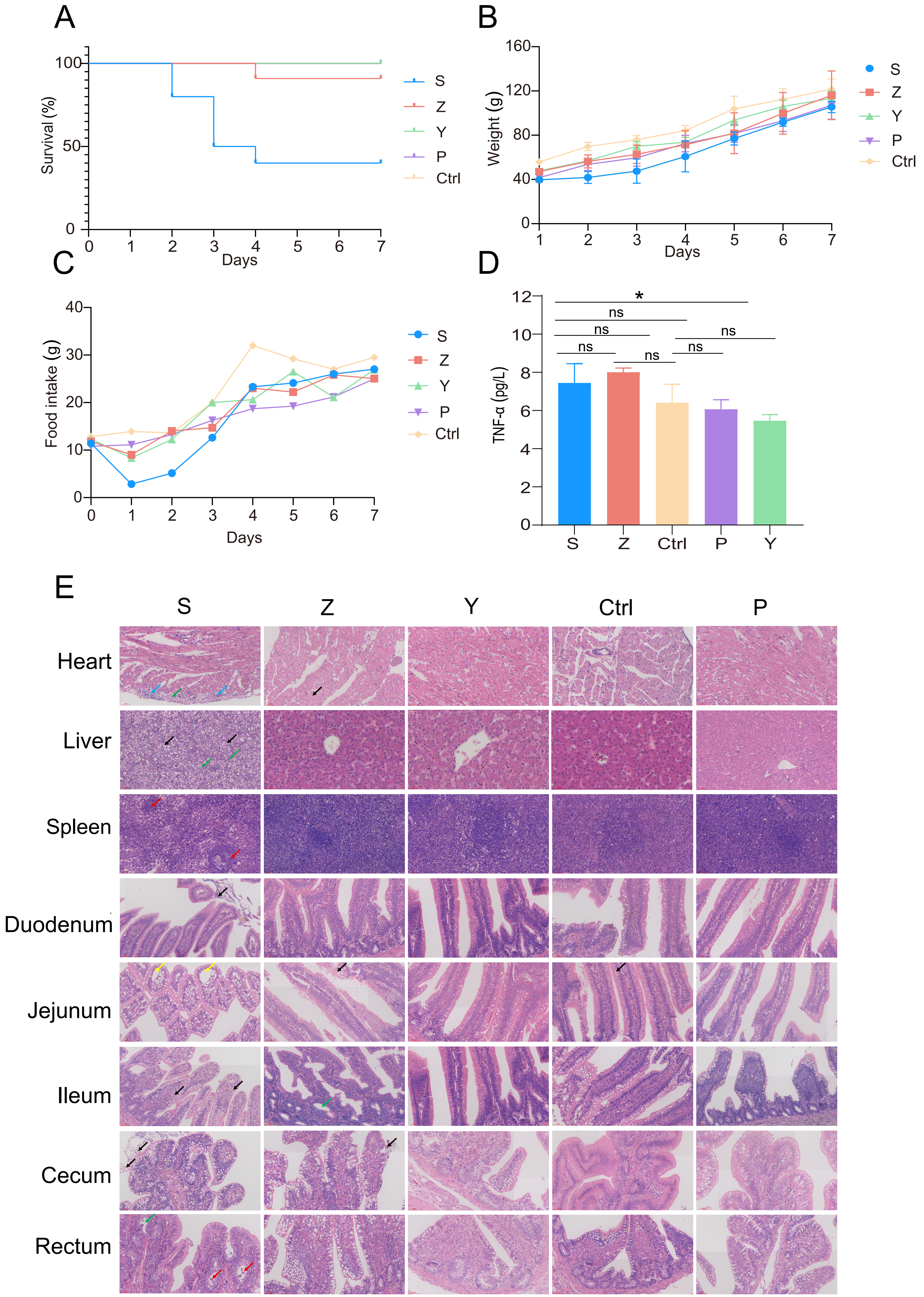

A
Survival (%)
100
50
0
0 1 2 3 4 5 6 7
Days
S
Z
Y
P
Ctrl
B
Weight (g)
160
120
80
40
0
1 2 3 4 5 6 7
Days
S
Z
Y
P
Ctrl
C
Food intake (g)
40
30
20
10
0
0 1 2 3 4 5 6 7
Days
S
Z
Y
P
Ctrl
D
TNF-α (pg/L)
12
10
8
6
4
2
0
*
ns
ns
ns
ns
ns
ns
S Z Ctrl P Y
E
S Z Y Ctrl P
Heart
Liver
Spleen
Duodenum
Jejunum
Ileum
Cecum
Rectum
